# Increased glymphatic system activity in migraine chronification by diffusion tensor image analysis along the perivascular space

**DOI:** 10.1186/s10194-023-01673-3

**Published:** 2023-11-06

**Authors:** Xue Zhang, Wei Wang, Xiaoyan Bai, Xueyan Zhang, Ziyu Yuan, Bingjie Jiao, Yingkui Zhang, Zhiye Li, Peng Zhang, Hefei Tang, Yaqing Zhang, Xueying Yu, Ruiliang Bai, Yonggang Wang, Binbin Sui

**Affiliations:** 1 Tiantan Neuroimaging Center for Excellence, China National Clinical Research Center for Neurological Diseases, Beijing Tiantan Hospital and Beijing Neurosurgical Institute, Capital Medical University, No.119 South Fourth Ring West Road, Fengtai District, Beijing, 100070 China; 2 Department of Neuroradiology, Beijing Neurosurgical Institute, Capital Medical University, Beijing, China; 3 Department of Radiology, Beijing Tiantan Hospital, Capital Medical University, Beijing, China; 4 Headache Center, Department of Neurology, Beijing Tiantan Hospital, Capital Medical University, No.119 South Fourth Ring West Road, Fengtai District, Beijing, China; 5 Department of Neurology, The First Affiliated Hospital of Zhengzhou University, Zhengzhou, China; 6 Key Laboratory of Biomedical Engineering of Education Ministry, College of Biomedical Engineering and Instrument Science, Zhejiang University, Hangzhou, China; 7 Department of Physical Medicine and Rehabilitation, School of Medicine of the Affiliated Sir Run Shumen Shaw Hospital and Interdisciplinary Institute of Neuroscience and Technology, Zhejiang University, Hangzhou, China; 8 MOE Frontier Science Center for Brain Science and Brain-machine Integration, School of Brain Science and Brain Medicine, Zhejiang University, Hangzhou, China

**Keywords:** Chronic migraine, Transformation, Glymphatic system, Diffusion tensor image, Magnetic resonance imaging

## Abstract

**Background:**

Preliminary evidence suggests that several headache disorders may be associated with glymphatic dysfunction. However, no studies have been conducted to examine the glymphatic activity in migraine chronification.

**Purposes:**

To investigate the glymphatic activity of migraine chronification in patients with episodic migraine (EM) and chronic migraine (CM) using the diffusion tensor image analysis along the perivascular space (DTI-ALPS) method.

**Methods:**

In this cross-sectional study, patients with EM, CM, and healthy controls (HCs) were included. All participants underwent a standard brain magnetic resonance imaging (MRI) examination. Bilateral DTI-ALPS indexes were calculated for all participants and compared among EM, CM, and HC groups. Correlations between the DTI-ALPS index and clinical characteristics were analyzed.

**Results:**

A total of 32 patients with EM, 24 patients with CM, and 41 age- and sex-matched HCs were included in the analysis. Significant differences were found in the right DTI-ALPS index among the three groups (*p* = 0.011), with CM showing significantly higher values than EM (*p* = 0.033) and HCs (*p* = 0.015). The right DTI-ALPS index of CM group was significantly higher than the left DTI-ALPS index (*p* = 0.005). And the headache intensity was correlated to DTI-ALPS index both in the left hemisphere (*r* = 0.371, *p* = 0.011) and in the right hemisphere (*r* = 0.307, *p* = 0.038), but there were no correlations after Bonferroni correction.

**Conclusions:**

Glymphatic system activity is shown to be increased instead of impaired during migraine chronification. The mechanism behind this observation suggests that increased glymphatic activity is more likely to be a concomitant phenomenon of altered vascular reactivity associated with migraine pathophysiology rather than a risk factor of migraine chronification.

**Supplementary Information:**

The online version contains supplementary material available at 10.1186/s10194-023-01673-3.

## Background

Migraine is a complex neurovascular disorder that affects approximately 1 billion people worldwide [1]. Its widespread prevalence and associated disability have led to it being the leading cause of disability in people younger than 50 years worldwide, especially in women [2]. Depending on the frequency of headache attacks per month, it can be divided into episodic migraine (EM) and chronic migraine (CM). The prevalence of CM is estimated to be 1.4-2.2% in the general population [3]. Approximately 3% of patients with EM progress to CM [4], with clinically progressive manifestations characterized by increasing frequency of headache attacks, which is identified as migraine chronification. Compared to EM, CM has a greater adverse effect on socioeconomic function and health-related quality of life [4]. Various mechanisms may promote the transformation from EM to CM. Central sensitization has been suggested as a pathophysiological mechanism underlying migraine chronification; in addition, dysfunction of the descending pain-modulating network, alterations in trigeminal and autonomic system function have also been discovered during the development of CM [4]. Nevertheless, pathophysiology of CM or the exact mechanisms underlying the transformation from EM to CM are still not entirely understood. A full understanding of the pathophysiological mechanisms underpinning the migraine chronification is essential to prevent the progression or reverse the transformation.

Recent evidence has identified a ‘waste clearance’ system in the central nervous system, defined as the glymphatic system, where the movement and interchange of cerebrospinal fluid (CSF) and interstitial fluid (ISF) along the perivascular space contribute to the transport and elimination of metabolic products [5]. Arterial pulsation, vasomotion and many physiological factors, including respiration, heart rate, and intracranial pressure, regulate the fluid transport of glymphatic system [6]. Impacts on any part of the glymphatic pathway of CSF inflow, CSF-ISF exchange, and ISF efflux, may ultimately affect the function of glymphatic system [7]. Preliminary studies have suggested a potential link between headache disorders and the glymphatic system [8]. Migraine is a neurovascular disorder involving the activation of trigeminovascular system [9]. Owing to the coupling relationship between cerebral blood flow and glymphatic system [10], glymphatic function in migraineurs may be affected by vascular dysfunction. An experimental study first revealed the neuronal event accompanied by migraine aura caused the temporary closure of perivascular space and impaired the glymphatic flow [11]. Afterwards, a pilot study has evaluated the glymphatic function in migraine and reported normal glymphatic system function in patients with migraine [12]. However, this study did not investigate whether glymphatic function changes during the migraine chronification. With headache attacks of increasing frequency, it remains unclear whether changes in glymphatic function occur during migraine chronification. Against the background, we hypothesize that glymphatic dysfunction during the migraine chronification, may be a risk factor underlying the transformation from EM to CM.

Diffusion tensor image analysis along the perivascular space (DTI-ALPS) method provided an opportunity to noninvasively investigate the glymphatic system in humans [13]. This method allows nearly independent analysis of the water diffusivity along the perivascular space by taking advantage of the perpendicular conditions of veins (x-axis) and domain fibers (y-axis and z-axis) at the level of lateral ventricle body, and thus reflecting the glymphatic system activity [13]. DTI-ALPS index showed a strong correlation with the glymphatic measurement of intrathecal contrast administration method [14]. Currently, the MRI technique has been widely used to evaluate glymphatic system activity in humans, and it has been demonstrated to be a reliable and practical method with good test–retest reproducibility and robustness [15].

The purpose of this study was to evaluate the activity of glymphatic system in patients with EM and CM by application of DTI-ALPS index to determine the potential changes in glymphatic activity underlying the migraine transformation, and analyzed the relationship between the DTI-ALPS index and clinical characteristics of migraine. Recognition of the associated factors underlying migraine transformation may help us to discover new disease markers or even future treatment targets.

## Methods

### Study design

This was a cross-sectional study based on the ongoing China HeadAche DIsorders RegiStry Study (CHAIRS, trial registration: NCT05334927). This study was approved by the Ethics Committee of Beijing Tiantan Hospital (no.KY2022-044). Each subject consented to participate in this study and signed informed consent.

### Participants

In this study, 63 patients with migraine (35 with EM and 28 with CM) and 45 healthy controls (HCs) were enrolled from the headache center of Beijing Tiantan Hospital and local communities between October 2020 and November 2022. Patients were diagnosed with EM and CM according to the International Classification of Headache Diseases, 3rd edition (ICHD-3) [16]. All patients had migraine without aura and without medication overuse. None of the patients was taking any migraine preventive medications. And they did not have migraine attacks or take any acute medications 24 h before magnetic resonance imaging (MRI) acquisition, to avoid the possible influences of analgesic on the glymphatic system obscuring the potential changes in glymphatic activity caused by migraine itself. All participants had no other headache disorders, no medical, neurological, or psychiatric conditions, and they were required to be free of major cardiovascular disorders since this may impair cardiovascular impulses in the human brain, thereby impacting glymphatic flow [17]. The participants without contraindications were allowed to perform MRI examinations (e.g., claustrophobia, MR-unsafe implants, pregnancy, or breastfeeding). Participants were excluded if they (1) had significant brain lesions detected on the MRI (e.g., lacunas, white matter hyperintensity with Fazekas score > 1); (2) did not complete the MRI examination; (3) had inadequate-quality images useless for data analysis.

Demographic data and clinical features of migraine were recorded. Neuropsychological assessments were administered by an experienced neurologist. Headache intensity was evaluated by Visual analogue scale (VAS). The Migraine Disability Assessment (MIDAS) [18] questionnaire and the Headache Impact Test-6 (HIT-6) [19] were given to measure migraine-related disability and adverse headache impact. The Patient Health Questionnaire-9 (PHQ-9) [20], the Generalized Anxiety Disorder Questionnaire-7 (GAD-7) [21], and the Pittsburgh Sleep Quality Index (PSQI) [22] were used to evaluate depression, anxiety, and sleep quality, respectively. Higher scores of MIDAS, HIT-6, PHQ-9, GAD-7, and PSQI correspond to higher degrees of disability, more adverse impact, anxiety, depression, and poorer sleep, respectively. A score of ≥ 10 on the PHQ-9 indicates depression [20], while a score of ≥ 10 on the GAD-7 indicates generalized anxiety disorder [21]. The Chinese version of the PSQI defines poor sleep quality as a PSQI score > 7 [23].

### MRI acquisition

All MRI examinations were performed on a 3.0T MRI scanner (Signa Premier, GE Healthcare, Waukesha, WI) using a 48-channel brain phased array coil. All participants underwent the standard brain MRI protocol between 9 a.m. and 5 p.m., and they were instructed to keep stable, stay awake and relax during the scans. All the patients were in the interictal state at the time of the MRI scans (there were no headache attacks during MRI scan nor 24 h before the scan). The MRI protocol included sagittal 3D T1-weighted magnetization-prepared rapid gradient-echo (MP-RAGE), sagittal 3D fluid-attenuated inversion recovery (FLAIR), axial T2-weighted image, and diffusion tensor image (DTI). DTI was performed with the following parameters: repetition time = 5258 ms, echo time = 85 ms, acquisition matrix = 104 × 104, field of view = 208 × 208 mm^2^, slice thickness = 2 mm, flip angle = 90°, gradient direction = 108, diffusion sensitivity coefficient (b) = 0, 1000 s/mm2. The acquisition time was 14 min and 40 s.

### Measurement of DTI-ALPS index

**Fig. 1:**
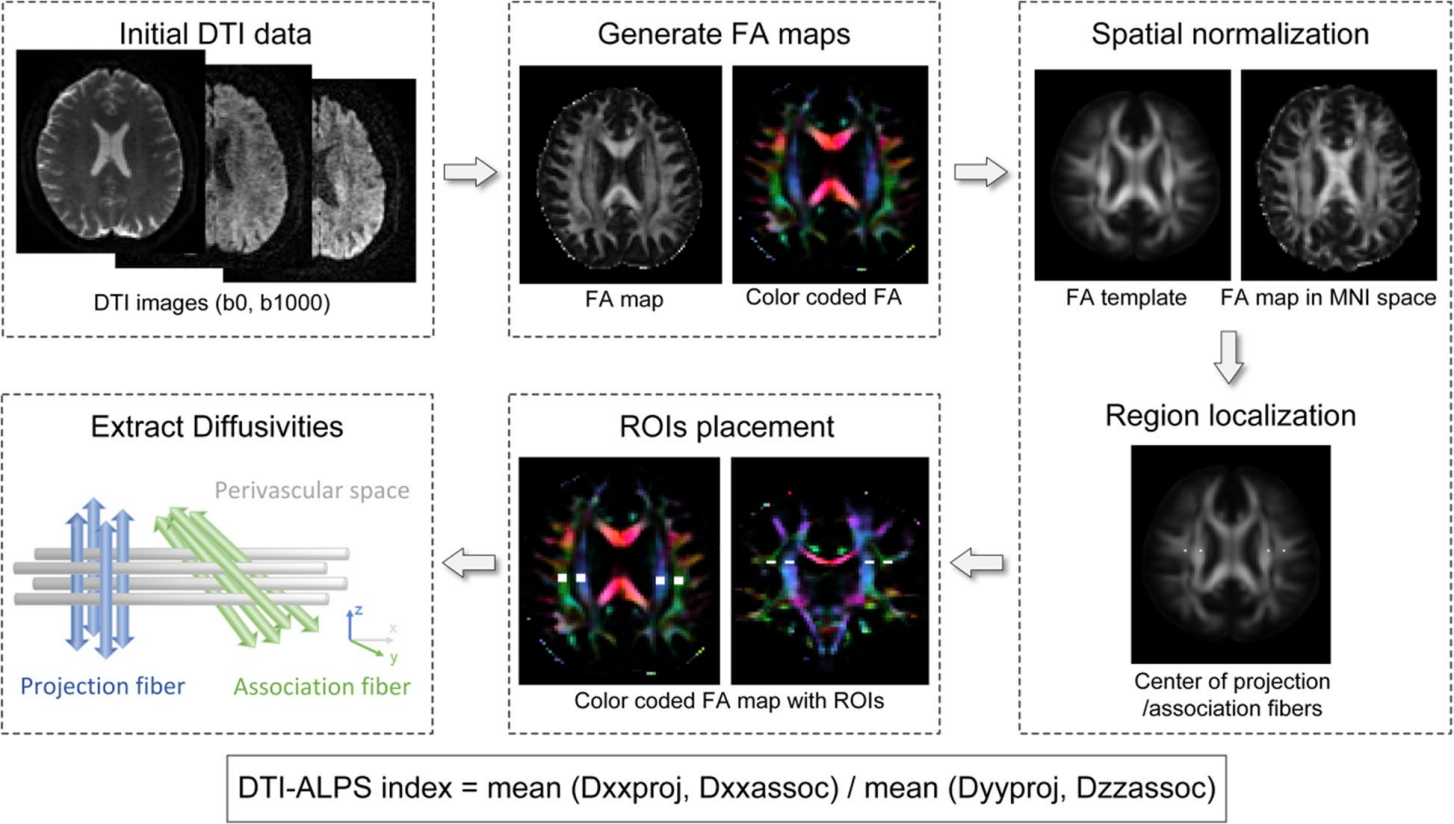
Flow diagram of the MRI method for the measurement of the DTI-ALPS index. DTI data were preprocessed to generate FA maps and color-coded FA maps, and these images were normalized to a standard template where the location of the ROIs were initially determined. Subsequently, four 6 × 6 mm^2^ ROIs were placed according to identified positions. Diffusivities in the directions of the x-, y-, and z-axis were recorded, and the DTI-ALPS index was calculated. DTI-ALPS, diffusion tensor image analysis along the perivascular space; FA, fractional anisotropy; DTI, Diffusion tensor image; ROI, region of interest

DTI-ALPS index was calculated in the same way as previously described [13, 14]. The method is described in Fig. 1. Firstly, DTI imaging underwent preprocessing steps, including the subject motion, eddy current correction, and skull stripping, to generate fractional anisotropy (FA) and color-coded FA maps using the Tortoise software package (National Institutes of Health, Bethesda, MD, v3.1.1) [24, 25]. For each subject, the FA map was registered to the FA template (https://neurovault.org/images/1406/) using Advanced Normalization Tools (ANTs, https://stnava.github.io/ANTs/). All transformations were visually checked for proper co-registration. Subsequently, we selected four 6 × 6 mm^2^ rectangular regions of interest (ROIs) at the FA template: two regions were drawn over the area of projection fibers (ROIproj) with the major fiber running along the z-axis, and the other two regions over the area of association fibers (ROIassoc) with the major fibers running along the y-axis in the bilateral hemispheres according to the previous method [14], to minimize the variance in the measurement of DTI-ALPS index induced by different regions. And then four ROIs were transferred to the individual FA color map by applying the inverse transformation matrix previously obtained through the FA map to the FA template. Visual inspections were applied to ensure the proper location of ROIs on their corresponding colored fibers that only blue voxels were included in the ROIproj and only green voxels in the ROIassoc. Finally, diffusivities along the x-axis (Dxx), y-axis (Dyy), and z-axis (Dzz) were extracted for each ROI.

DTI-ALPS was calculated as the ratio of diffusivities perpendicular to fiber bundles and parallel to perivascular space (Dxxproj and Dxxassoc) over diffusivities perpendicular to fiber bundles and perpendicular to perivascular space (Dyyproj and Dzzassoc) [13]. DTI-ALPS index in the left hemisphere (left DTI-ALPS index) and right hemisphere (right DTI-ALPS index) were calculated respectively using the following formula. DTI-ALPS index is a dimensionless quantity.

$$DTI-ALPS\;index = mean (Dxxproj,\;Dxxassoc)/mean (Dyyproj,\;Dzzassoc)$$

### Statistical analyses

The sample size was based on the available data and previous literature. A sample size of 97 cases (41 HCs, 32 EM, and 24 CM) would provide 85% power to reject the null hypothesis equal means when the group means are 1.63, 1.64, 1.77 with standard deviations of 0.18, 0.17, 0.23 at a two-sided alpha of 0.05. All statistical analyses were performed using SPSS software (version 25.0, IBM-SPSS, Chicago, IL, USA). The Shapiro–Wilk test was used to test the normality of continuous variables. Continuous variables were expressed as mean ± standard deviation (SD) or median with interquartile range (IQR). Categorical variables were presented as frequencies and percentages.

Differences in the demographic and clinical characteristics between the groups were analyzed using independent-samples t-test or Mann–Whitney U test depending on whether they followed a normal distribution for continuous variables; Chi-square test was used for categorical variables. Comparisons of the DTI-ALPS index among the HCs, EM and CM groups were performed using one-way analysis of variance (ANOVA) followed by post hoc multi-comparisons with Bonferroni test. Statistical significance was set at a two-tailed *p* < 0.05. To analyze the correlation between DTI-ALPS index and clinical characteristics, taking into account the influence of age and sex on DTI-ALPS index [26], we performed the correlation analysis adjusted for age and sex. Multiple corrections were applied when correlation analysis between the DTI-ALPS index and clinical characteristics (Bonferroni correction, *p* < 0.0025[0.05/20]) was conducted.

## Results

### Demographics and clinical characteristics

**Fig. 2:**
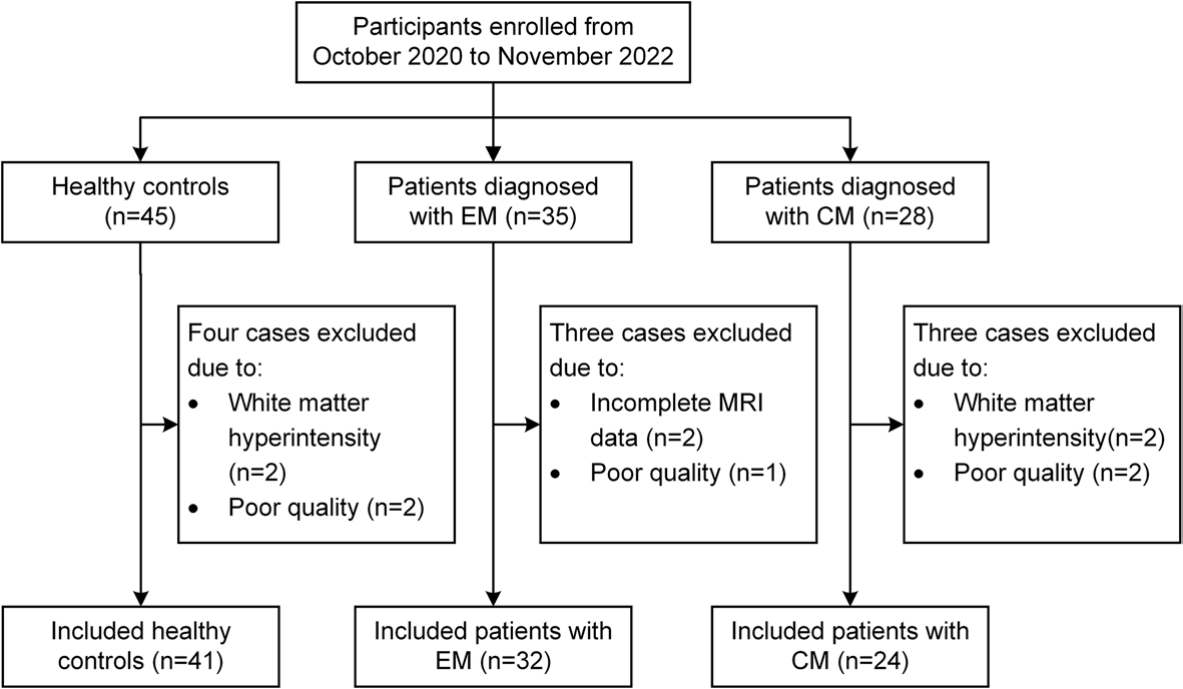
Flowchart of participants’ enrollment in this study. EM, episodic migraine; CM, chronic migraine

Thirty-five patients with EM, 28 patients with CM, and 45 age-and sex-matched HCs were enrolled in this study. After excluding 3 patients with EM, 4 patients with CM, and 4 HCs due to the white matter hyperintensity, incomplete MRI data and poor imaging quality (Fig. 2), the final analysis consisted of 32 patients with EM (9 males, 23 females; age, 34.8 ± 15.4 years), 24 patients with CM (9 males, 15 females; age, 34.8 ± 16.8 years) and 41 HCs (16 males, 25 females; age, 36.0 ± 10.9 years). The demographic and clinical data of all subjects are shown in Table 1. There was no significant difference in age and sex among the three groups. Patients with CM had significantly more headache attacks than patients with EM (*p* < 0.001). Three quarters of patients with migraine have headache attacks involving both hemispheres.

**Table 1:** Demographic and clinical features of healthy controls, patients with EM and CM

	HC (*n* = 41)	EM (*n* = 32)	CM (*n* = 24)	*p* value^a^	*p* value^b^
Age (years)	36.0 ± 10.9	34.8 ± 15.4	34.8 ± 16.8	0.924	0.983
Sex (male/female)	16/25	9/23	9/15	0.600	0.457
BMI (kg/m^2^)	22.4 ± 2.7	23.3 ± 4.0	22.8 ± 3.3	0.538	0.604
Disease duration (years)	−	11.5 (4.0–20.0)	10.0 (5.3–22.3)	−	0.666
Age of onset (years)	−	22.0 (13.0–32.9)	17.0 (12.3–26.8)	−	0.393
Headache intensity (VAS)	−	7.0 (5.0–8.0)	7.0 (6.0–8.0)	−	0.433
Attack frequency (days/month)	−	6.5 (4.0–10.0)	19.0 (15.0–30.0)	−	< 0.001
Side of Headache					
Bilateral, n (%)	−	24 (75.0%)	18 (75.0%)	−	−
Unilateral, n (%)	−	8 (25.0%)	6 (25.0%)	−	−
Left-sided, n (%)	−	5 (15.6%)	4 (16.7%)	−	−
Right-sided, n (%)	−	3 (9.4%)	2 (8.3%)	−	−

*HC *Healthy control, *EM *Episodic migraine, *CM *Chronic migraine, *BMI *Body mass index, *VAS *Visual analogue scale

^a^ *p* value among three groups by analysis of variance; ^b^ *p* value for EM vs. CM by t-test

The MIDAS, HIT-6, PHQ-9, GAD-7, and PSQI scores were available to 25 patients with EM and 23 patients with CM. HCs didn’t administer the scales. Compared to patients with EM, those with CM had more severe disability (*p* < 0.001), severe degree of depression (*p* < 0.001) and anxiety (*p* = 0.012). Patients with CM had significantly higher rates of depression than patients with EM, with a prevalence rate of 56.5% versus 16.0% (*p* = 0.003) (Table 2).

**Table 2:** Neuropsychology tests of patients with migraine

Questionnaires and scales	Episodic migraine (*n* = 25)	Chronic migraine (*n* = 23)	*p* value
MIDAS score	48.0 (18.0–60.0)	95.0 (58.0–179.0)	< 0.001
HIT-6 score	64.2 ± 6.8	68.0 ± 5.5	0.043
PHQ-9 score	5.0 (1.0−7.0)	12.0 (6.0−16.0)	< 0.001
GAD-7 score	3.0 (1.0–8.5)	9.0 (5.0−13.0)	0.012
PSQI score	8.5 ± 4.8	9.1 ± 4.7	0.640
Depression, n (%)	4 (16.0%)	13 (56.5%)	0.003
Generalized anxiety disorder, n (%)	6 (24.0%)	11 (47.8%)	0.085
Poor sleep quality, n (%)	13 (52.0%)	14 (60.9%)	0.536

Depression was defined as PHQ-9 score ≥ 10; Generalized anxiety disorder was defined as GAD-7 score ≥ 10; Poor sleep quality was defined as PSQI score > 7

*MIDAS *Migraine Disability Assessment, *HIT-6 *Headache Impact Test-6, *PHQ-9 *Patient Health Questionnaire-9, *GAD-7 *Generalized Anxiety Disorder-7, *PSQI *Pittsburgh Sleep Quality Index

### Comparisons of DTI-ALPS index in migraine chronification

**Fig. 3:**
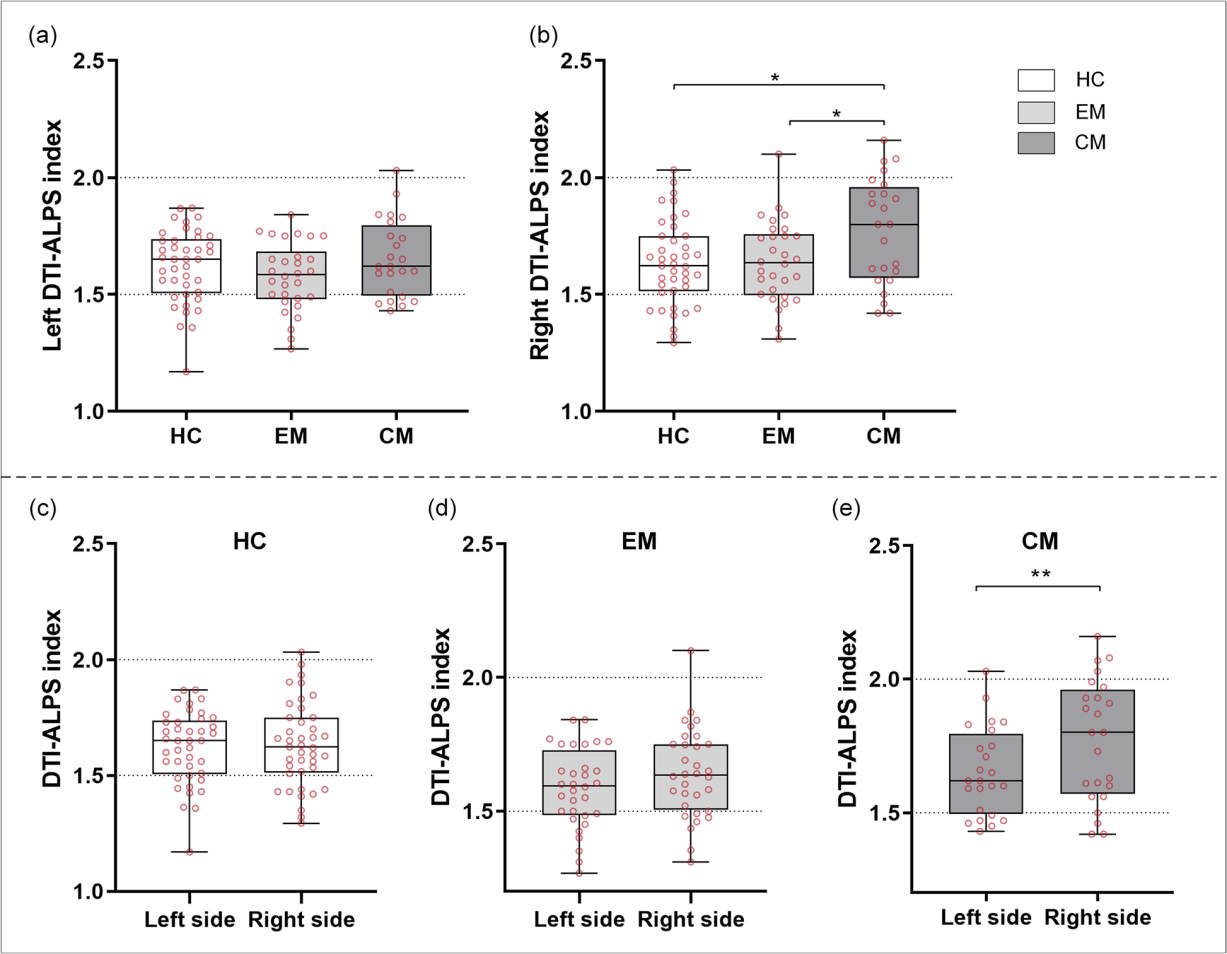
Differences in DTI-ALPS index among HC, EM and CM groups. **a** There was no significant difference was observed in left DTI-ALPS index among HC, EM and CM groups. **b** The right DTI-ALPS index in patients with CM was significantly higher than HCs (*p* = 0.015) and EM (*p* = 0.033). **c**-**e** Comparisons of left and right DTI-ALPS index among the three groups. The right DTI-ALPS index of patients with CM was significantly higher than left DTI-ALPS index (*p* = 0.005). No significant difference was observed between left and right DTI-ALPS index in HCs and EM. DTI-ALPS, diffusion tensor image analysis along the perivascular space; HC, healthy control; EM, episodic migraine; CM, chronic migraine. * *p* < 0.05, ** *p* < 0.01.

Significant differences in the right DTI-ALPS index were observed among the three groups (*p* = 0.011), with the patients with CM showing the highest values. Post hoc analyses revealed that patients with CM had significantly higher right DTI-ALPS index than HC (1.77 ± 0.23 vs. 1.63 ± 0.18, *p* = 0.015, mean difference (MD) [95% confidence interval (CI)] = 0.140 [0.021–0.258]), and EM (1.77 ± 0.23 vs. 1.64 ± 0.17, *p* = 0.033, MD [95% CI] = 0.133 [0.008–0.257), but there was no difference between HC and EM groups. As for the left DTI-ALPS index, there were no significant difference among the three groups (*p* = 0.260) (Fig. 3).

In patients with CM, the right DTI-ALPS index was significantly higher than the left DTI-ALPS index (1.77 ± 0.23 vs. 1.65 ± 0.16, *p* = 0.005, MD [95%CI] = 0.118 [0.040–0.197). However, there was no significant difference between the left DTI-ALPS and right DTI-ALPS index in patients with EM (1.64 ± 0.17 vs. 1.59 ± 0.15, *p* = 0.110) (Fig. 3).

### Correlation between DTI-ALPS index and migraine characteristics

To avoid the effect of age and sex on the DTI-ALPS index [26], we performed correlation analyses after adjustment for age and sex. The results showed that severe headache intensity (VAS score) of patients with migraine was significantly correlated with the DTI-ALPS index in both the left hemisphere (*r* = 0.371, *p* = 0.011) and the right hemisphere (*r* = 0.307, *p* = 0.038) (Fig. S1) But, after Bonferroni correction, there were no significant correlations between the DTI-ALPS index and headache intensity (Table 3); in addition, there was no any significant correlation between DTI-ALPS index and clinical characteristics in the EM and CM groups, respectively (Table S1).

**Table 3:** Correlations between DTI-ALPS index and clinical characteristics of migraine

Variables	Left DTI-ALPS index		Right DTI-ALPS index	
	*r*	*p*	*r*	*p*
BMI (kg/m^2^)	-0.083	0.551	-0.014	0.922
Disease duration (years)	0.189	0.209	0.181	0.229
Age of onset (years)	-0.190	0.206	-0.181	0.229
Headache intensity (VAS)	0.371	0.011	0.307	0.038
Attacks frequency (days/month)	0.081	0.592	0.137	0.363
MIDAS score^a^	0.165	0.272	0.125	0.408
HIT-6 score^a^	-0.094	0.534	0.009	0.955
PHQ-9 score^a^	0.243	0.104	0.107	0.479
GAD-7 score^a^	0.192	0.201	0.192	0.201
PSQI score^a^	0.013	0.934	0.094	0.533

Statistical significance: *p* < 0.0025 (Bonferroni correction)

*DTI-ALPS *Diffusion tensor image analysis along the perivascular space, *EM *Episodic migraine, *CM *Chronic migraine, *BMI *Body mass index, *MIDAS *Migraine Disability Assessment, *HIT-6 *Headache Impact Test-6, *PHQ-9 *Patient Health Questionnaire-9, *GAD-7 *Generalized Anxiety Disorder-7; PSQI, Pittsburgh Sleep Quality Index

^a^Available to 48 patients with migraine

## Discussion

In this study, we investigated the alterations in the activity of glymphatic system during the migraine transformation from EM to CM by means of DTI-ALPS method. Our results revealed that patients with CM had a significantly higher DTI-ALPS index in the right hemisphere compared with EM and HCs, indicating an abnormality of the glymphatic system in migraine chronification, and that the abnormality was right-dominant. These intriguing findings hint at lateralized changes in the glymphatic activity during migraine chronification.

In the glymphatic framework, cardiac-driven motion of vessel wall drives the bulk flow of CSF in the direction of blood flow, into deep periarterial spaces, and then drives convective ISF flow towards and into the perivenous spaces, finally this process successfully facilitates the drainage of interstitial solutes from the brain to meningeal and cervical lymphatic drainage vessels [5, 27, 28]. Previous studies have found the significant lower DTI-ALPS index in aging [26], Alzheimer’s disease [13], Parkinson’s disease [29], and multiple sclerosis [30], indicating an reduced glymphatic activity and impaired glymphatic clearance function. In contrast, the higher DTI-ALPS index observed in patients with CM in the present study may be indicative of unimpaired or even improved glymphatic system activity of migraine chronification.

Migraine is a neurovascular disorder involving the activation of trigeminovascular system, with the release of vascular neuropeptides, most especially calcitonin gene-related peptide (CGRP), and dysfunction of vascular tone regulation [9]. CGRP has been considered as a central role in migraine pathogenesis [31]. The release of CGRP induces the vasodilation of meninges and intracranial arteries and local inflammatory response giving rise to the typical throbbing or pulsating migraine pain [32]. These vascular responses induced by CGRP and as a consequence vasodilation may affect the activity of glymphatic system. It is well known that alcohol consumption is considered to be a trigger for migraine; at the same time, previous studies have shown that low-dose alcohol, acting as a potent vasodilator, can promote the glymphatic flow [33, 34], by the activation of endothelial specific nitric oxide synthase (eNOS) and generation of nitric oxide (NO), which increasing the interactive reactivity of vascular endothelial and smooth muscle cells, thus regulating the enhanced glymphatic clearance of metabolites [33]. Considering that CGRP can elicit pathophysiologic responses, e.g., neuronal excitability, neurotransmitter release, NO production, and vasodilation [35], with the final effects similar to those of low-dose alcohol, we speculate that increased glymphatic system function in patients with CM observed in the present study may be related to the downstream vascular response induced by the release of CGRP.

During migraine attack, increased release of CGRP in the jugular venous blood has been reported [36]. Furthermore, the peripheral CGRP concentrations in patients with CM were consistently elevated in the interictal period [37], which suggests altered interictal activity of the trigeminal vascular system in CM. During the development of CM, persistent release of CGRP is thought to induce central sensitization and migraine chronification. We investigated the glymphatic system activity in the interictal phase in patients with migraine, and found that the interictal DTI-ALPS index was significantly higher in patients with CM than HCs and EM. These results support our proposal that increased activity of glymphatic system during migraine chronification may be a concomitant response to the persistent release of CGRP. The effect of CGRP in regulating glymphatic system, however, could not be directly examined in this study because data on CGRP concentrations were not available.

Additionally, arterial pulsation is a significant driving force of perivascular fluid dynamics. Experimental increase of vascular wall pulsatility using a beta-adrenergic agonist accelerated the rate of paravascular CSF influx into brain parenchyma [38]. Vasomotion of the vessel, a spontaneous rhythmic cerebral vasoconstriction and dilation of the vascular smooth muscle cells' motions, is another efficient driver of glymphatic circulation. A previous study has reported that the clearance rate of fluorescent tracer is also increased in mice brain when amplitude of vasomotion physiologically increased by functional hyperemia [39, 40]; on the other hand, the reduction of cerebral blood flow lessens arterial wall pulsatility, thus attenuating CSF-ISF exchange in the brain [38]. These results suggest a functional connection between glymphatic system and cerebral hemodynamics. Moreover, cerebral hyperperfusion patterns in migraineurs have been reported in several studies [41–43]. Regional cerebral hyperperfusion may regulate glymphatic system activity by influencing vasomotion and arterial pulsatility in patients with migraine. Future studies incorporating an analysis of cerebral hemodynamics and glymphatic system in migraine will further explain the mechanisms underlying the increased glymphatic activity in patients with CM.

It appears that increased activity of glymphatic system in CM is more of a concomitant phenomenon of altered vascular reactivity induced by the persistent release of CGRP rather than a causative factor for the migraine chronification. But it is still worthwhile to explore whether increased glymphatic system activity in patients with CM can serve as a protective factor against brain aging and neurodegenerative disorders.

In our study, although chronic migraineurs have increased glymphatic system activity than patients with EM, no correlation was found between glymphatic system activity and migraine attack frequency. This result was consistent with a previous study, which investigated the glymphatic system function of patients with migraine with and without aura, and similarly they did not find the correlation between the DTI-ALPS index and migraine attack frequency [12]. Although difference in migraine attack frequency is a clinical criterion for distinguishing CM from EM, the transformation from EM to CM is often accompanied by a variety of risk factors, such as, overuse of acute migraine medication, ineffective acute treatment, obesity, depression, and stressful life events [4]. Furthermore, it has been demonstrated that multimodal and domain-general differences between CM and EM goes beyond a differentiation based on the days of migraine per month [44]. In our study, the lack of correlation may suggest that the increased frequency of migraine attacks is not directly responsible for the increased activity of the glymphatic system in CM patients. As we discussed above, these differences in glymphatic system activity seem to be due to the other factors or pathological changes occurring in the migraine chronification process.

In the present study, 25% of patients presented with only unilateral migraine attacks, whereas a previous study revealed that lateralized, predominantly right-sided headache was suffered by about half of the 188 chronic headache patients. Differences in the study populations may have contributed to the differences in results. Additionally, in our study, patients with bilateral involvement included individuals who were involved bilateral hemispheres during each attack as well as during different migraine episodes, which may also contribute to the higher rate of migraineurs with bilateral headaches. However, the prominent alterations in glymphatic activity of patients with CM were predominantly lateralized on the right hemisphere. It is hard to directly explained the abnormality in glymphatic system in terms of involved side of migraine attacks. We venture to speculate that the right hemisphere may take precedence in the pathophysiological development of migraine. In addition to the glymphatic system function, the lateralized manifestation of headaches has also been reported in several previous studies [42, 43, 45–47]. The changes in the functional connectivity between the right thalamus and several cortex regions associated with pain modulation as well as pain coding in migraine have also been demonstrated by functional magnetic resonance imaging studies [46]. A magnetic resonance spectroscopy study revealed that the right thalamus of migraineurs had increased level of glutamate/glutamine [47]. Also, abnormal cerebral perfusion in right brain regions has been observed in patients with EM [42, 43]. Lateralized presentations of headache in terms of function, metabolism, and cerebral perfusion support that right dominance might be a feature of migraine, but longitudinal studies are needed to confirm this assumption.

This study has some limitations. First, our study is a cross-sectional study with a small sample size, thus a large sample cohort is needed to validate our findings. Second, the DTI-ALPS index represents diffusivity along the perivenous space at the slice of lateral ventricle body, which may only reflect partial function of the whole glymphatic system, and thus the correlation between DTI-ALPS index and glymphatic activity should be interpreted cautiously. Third, this study proposed the hypothesis of the role of vascular reactivity induced by CGRP in the increased glymphatic activity, but the data on CGRP were not obtained in the present study. Future studies will validate the relationships between altered glymphatic system function, CGRP concentration and cerebral hemodynamics in a longitudinal migraine cohort.

## Conclusion

The higher DTI-ALPS index in patients with CM than HCs and EM indicates the increased activity of glymphatic system during the migraine chronificiation. The mechanism behind this observation suggests that increased glymphatic activity is more likely to be a concomitant phenomenon of altered vascular reactivity associated with migraine pathophysiology rather than a risk factor of migraine transformation. Further studies are needed to confirm the relationship between the cerebral hemodynamics induced by the vascular neuropeptides and improved glymphatic activity in chronic migraine.

### Supplementary Information

**Additional file 1: Figure S1.** Correlations between headache intensity and DTI-ALPS index both in the left hemisphere (a) and right hemisphere (b) before Bonferroni correction (*p *< 0.0025, statistical significance). DTI-ALPS, diffusion tensor image analysis along the perivascular space; VAS, Visual analogue scale. **Table S1.** Correlations between DTI-ALPS index and clinical characteristics of episodic migraine and chronic migraine adjusted for age and sex

## Data Availability

The datasets analysed during the current study are available from the corresponding author on reasonable request.

## Abbreviations

ANOVA: One-way analysis of variance
CI: Confidence interval
CM: Chronic migraine
CGRP: Calcitonin gene-related peptide
CSF: Cerebrospinal fluid
DTI-ALPS: Diffusion tensor image analysis along the perivascular space
EM: Episodic migraine
eNOS: Endothelial specific nitric oxide synthase
FA: Fractional anisotropy
GAD-7: Generalized Anxiety Disorder-7
HC: Healthy control
HIT-6: Headache Impact Test-6
ICHD-3: International Classification of Headache Diseases, 3rd edition
IQR: Interquartile range
ISF: Interstitial fluid
MD: Mean difference
MIDAS: Migraine Disability Assessment
MRI: Magnetic resonance imaging
NO: Nitric oxide
PHQ-9: Patient Health Questionnaire-9
PSQI: Pittsburgh Sleep Quality Index
ROI: Region of interest
SD: Standard deviation
VAS: Visual analogue scale
