# Supplementary material for: Increased glymphatic system activity in migraine chronification by diffusion tensor image analysis along the perivascular space
Source: J Headache Pain. 2023 Nov 6;24(1):147. doi: 10.1186/s10194-023-01673-3 (PMC10626803; doi:10.1186/s10194-023-01673-3)
Supplement: Supplementary file 1 — Additional file 1: Figure S1. Correlations between headache intensity and DTI-ALPS index both in the left hemisphere (a) and right hemisphere (b) before Bonferroni correction (p < 0.0025, statistical significance). DTI-ALPS, diffusion tensor image analysis along the perivascular space; VAS, Visual analogue scale. Table S1. Correlations between DTI-ALPS index and clinical characteristics of episodic migraine and chronic migraine adjusted for age and sex [file 10194_2023_1673_MOESM1_ESM.docx]

**

**

**Figure S1**. Correlations between headache intensity and DTI-ALPS index both in the left hemisphere (a) and right hemisphere (b) before Bonferroni correction (*p* < 0.0025, statistical significance). DTI-ALPS, diffusion tensor image analysis along the perivascular space; VAS, Visual analogue scale.

**Table S1**. Correlations between DTI-ALPS index and clinical characteristics of episodic migraine and chronic migraine adjusted for age and sex.

| Variables | Left DTI-ALPS index | | Right DTI-ALPS index | |
| --- | --- | --- | --- | --- |
|  | *r* | *p* | *r* | *p* |
| **Episodic migraine** |  |  |  |  |
| BMI (kg/m^2^) | 0.023 | 0914 | 0.045 | 0.837 |
| Disease duration (years) | 0.283 | 0.191 | 0.183 | 0.402 |
| Age of onset (years) | -0.287 | 0.184 | -0.186 | 0.397 |
| Headache intensity (VAS) | 0.396 | 0.062 | 0.433 | 0.039 |
| Attacks frequency (days/month) | 0.071 | 0.749 | -0.158 | 0.473 |
| MIDAS score † | 0.039 | 0.860 | -0.038 | 0.865 |
| HIT-6 score † | -0.130 | 0.554 | 0.092 | 0.676 |
| PHQ-9 score † | 0.014 | 0.948 | -0.182 | 0.406 |
| GAD-7 score † | 0.110 | 0.617 | -0.350 | 0.102 |
| PSQI score † | -0.126 | 0.567 | -0.087 | 0.693 |
| **Chronic migraine** |  |  |  |  |
| BMI (kg/m^2^) | -0.281 | 0.217 | -0.100 | 0.665 |
| Disease duration (years) | 0.088 | 0.705 | 0.137 | 0.555 |
| Age of onset (years) | -0.087 | 0.707 | -0.136 | 0.558 |
| Headache intensity (VAS) | 0.277 | 0.224 | 0.124 | 0.593 |
| Attacks frequency (days/month) | -0.388 | 0.082 | -0.183 | 0.427 |
| MIDAS score ‡ | 0.051 | 0.826 | -0.046 | 0.844 |
| HIT-6 score ‡ | -0.247 | 0.280 | -0.277 | 0.224 |
| PHQ-9 score ‡ | 0.254 | 0.266 | 0.032 | 0.891 |
| GAD-7 score ‡ | 0.128 | 0.581 | -0.064 | 0.783 |
| PSQI score ‡ | 0.121 | 0.602 | 0.228 | 0.319 |

Note: Statistical significance: *p* < 0.0025 (Bonferroni correction).

Abbreviations: DTI-ALPS, Diffusion tensor image analysis along the perivascular space; EM, episodic migraine; CM, chronic migraine; BMI, body mass index; MIDAS, Migraine Disability Assessment; HIT-6, Headache Impact Test-6; PHQ-9, Patient Health Questionnaire-9; GAD-7, Generalized Anxiety Disorder-7; PSQI, Pittsburgh Sleep Quality Index.

† Available to 25 patients with episodic migraine.

‡ Available to 23 patients with chronic migraine.
